# On the Investigation of Effective Factors on Higher Heating Value of Biodiesel: Robust Modeling and Data Assessments

**DOI:** 10.1155/2021/4814888

**Published:** 2021-07-12

**Authors:** Shicheng Wang, Wei Li, Issam Alruyemi

**Affiliations:** ^1^School of Economics and Management, Southwest Petroleum University, Chengdu, Sichuan 610500, China; ^2^Petroleum Engineering School, Southwest Petroleum University, Chengdu, Sichuan 610500, China; ^3^Fouman Faculty of Engineering, College of Engineering, University of Tehran, Fouman, Iran

## Abstract

Higher heating value (HHV) is one of the properties of biomass fuels which is essential in investigating their special characteristics and potentialities. In this paper, various techniques based on Gaussian process regression (GPR) were utilized to assess this value for biomass fuels, including several kernel functions, i.e., exponential, Matern, rational quadratic, and squared exponential. An extensive databank was collected from literature. The findings were compared, and the results indicated that Exponential-based model was more accurate, with the coefficient of regression (*R*^2^) of 0.961 and the mean relative error (% MRE) of 3.11 for total data. Compared to former models presented by previous researchers, the model proposed in this study showed a higher ability to predict output values. With various analyses, it can be concluded that the proposed method has a high rate of efficiency in assessing the HHV of various biomass.

## 1. Introduction

The use of fossil fuels has problems and disadvantages such as environmental pollution, asphaltene deposition, and limited resources [1–3]. There have been growing attempts at reducing the use of conventional fossil fuels and finding suitable replacements to use in a world with an ever-increasing population and industrial expansion, a compromised environment, and steadily depleting energy sources. Among these alternatives, biomass has become of particular interest due to its carbon neutrality and ease in being processed (e.g., chemically, thermally, and biochemically) to produce energy [4]. In recent years, coal-fired power stations have turned to use biomass to replace part of their fuel. This way, without needing to change any of their equipment, they can lower their use of coal and thus contribute to environmental and economic prosperity [5, 6].

Characteristics of biomass fuel, before being incorporated as a renewable source of energy, must be fully identified. Among these characteristics, the higher heating value is fundamental for allocating the feedstock for specific uses. The conventional method of measuring the HHV for liquid and solid fuel is adiabatic oxygen bomb calorimetry, which is, however, expensive and inefficient [7]. There are two methods of finding correlations for HHV: ultimate and proximate. The former is capable of identifying the composition of the fuel and its elements [8] but is more expensive than the latter method and cannot function without specific prior experiments. This has led to the widespread use of the proximate method of analysis [9]. This method works by first determining the changes in the enthalpy of products and reactants of a specific type of fuel. The procedure is not complicated but it takes a long time and requires equipment that might sometimes be unavailable. As a result, calculations are made by other empirical methods using the data from proximate or elemental analysis. Proximate analysis, which is simpler and faster, has more widespread use for measuring the HHV. From the gathered data, fixed carbon (FC), volatile matter (VM), and ash are the factors incorporated in calculations [10, 11].

In previous years, the use of artificial intelligence (AI) methods has many applications in various fields, and researchers have investigated complete and close analyses to develop empirical methods (which mostly involve linear and nonlinear models) to reliably approximate the HHV of different types of biomass fuel [12]. Despite the efforts to estimate biomass, the complications associated with its structure make understanding the relationship between HHV and the data from proximate or ultimate analysis problematic. As a result, attention has recently been turned to artificial intelligence and its high potential to solve complicated problems. Mesroghli et al. utilized ANN models to assess the HHV of coal [13]. Ghugare et al. assessed the HHV of solid biomass fuel utilizing MPL-ANN and GA-based models and used ultimate analysis to find correlation [14]. Another attempt at estimating HHV of biomass was undertaken by Hosseinpour et al. [15] using iterative neural network-adapted partial least squares. The data gathered by the proximate analysis were incorporated into an ANFIS model by Akkaya to estimate the heating value (HV) of biomass [16]. Uzun et al. experimented with various ANN structures to estimate the HHV of biomass [17]. Finally, Estiati et al. utilized ANN together with a few linear models [18].

The present study involves expanding models of estimating the HHV for biomass fuels to replace the ultimate analysis with the proximate analysis, which is both cheaper and faster. Innovative models are introduced based on Gaussian process regression modeling including four kernel functions, i.e., exponential, Matern, rational quadratic, and squared exponential. To design the models, the data regarding the HHV of various biomass were gathered from 382 studies. A comparison is drawn of these models with those studied and published in the past. The new models were further studied for their efficacy and usefulness in six types of biomass fuel.

## 2. Materials and Methods

### 2.1. Data Collection

The independent variables of volatile matter (VM), ash (A), and fixed carbon (FC) content on dry basis are the inputs in the present study. The output is the data regarding the HHV of biomass. Here, the aim is to find the most practical *y* or function *f* for the input data *x*_1_, *x*_2_, *x*_3_, i.e., FC, VM, and A, and *y* or function *f* indicates the HHV of biomass fuels.

The data from 382 proximate analyses regarding biomass and their HHVs were gathered from open literature. The data collected have been reported elsewhere [19]. The data regarding HHV were categorized into the following six groups:

1. Byproducts of fruits
2. Agri-wastes
3. Wood chips and/or tree species
4. Grasses, leaves, and fibrous materials
5. Other waste materials
6. Briquettes, charcoals, and pellets

Learning from literature, 30% of the data were randomly set apart as a test set to prevent overtraining [20]. Designing and training the nonlinear regression and AI models were performed using the remaining 70%. The test dataset helped examine the precision of the results and generalize the newly proposed models.

### 2.2. Gaussian Process Regression

To establish Gaussian process regression (GPR), it is required to select random training dataset *L* = {*x*_*L*,*i*_, *Y*_*L*,*i*_}_*i*=1_^*n*^ and testing dataset *T* = {*x*_*T*,*i*_, *Y*_*T*,*i*_}_*i*=1_^*n*^ from a particular distribution. The training dataset is employed to set the tuning parameters of the model [21, 22]. The testing dataset, which includes the excluded observations of the previous stage, is utilized to perform the approximate justification of the extended model. Also, *x* is the input variable, while *y* denotes the target variable. They are impacted by noise. The general form of GPR modeling is formulated as [22]:

(1) $$\begin{array}{c} y_{L,i}=f\left(x_{L,i}\right)+\varepsilon_{L,i},\;i=1.2.3\cdots \cdots n, \end{array}$$

in which *x*_*L*_ is the independent variable of the learning dataset, *y*_*L*_ is the learning dataset target, and *ε*^~^*N*(0, *σ*_noise_^2^*I*_*n*_) represents the observation noise of an independent Gaussian distribution (where *σ*_noise_^2^ stand for the noise variance, while *I*_noise_ represent the unit array variance). Then, the measured targets are connected to the function *f*(*x*) by using a Gaussian noise model [23, 24]. It is worth mentioning that *f* values are assumed to be random variables in the GP. Likewise,

(2) $$\begin{array}{c} y_{T,i}=f\left(x_{T,i}\right)+\varepsilon_{T,i},\;\;i=1.2.3\cdots \cdots ..n, \end{array}$$

in which *x*_*T*_ is the testing dataset independent variable, while *y*_*T*_ is the testing dataset target. Also, *f*(*x*) is a latent parameter and has a GP distribution with a mean of *m*(*x*) and covariance of *k*(*x*, *x*′) [23].

(3) $$\begin{array}{c} f\left(x_{T,i}\right)\sim Gp\left(m\left(x\right),K\left(x.x^{\prime }\right)\right). \end{array}$$

To specify the mean function *m*(*x*), one can utilize an explicit basis function, even though it would lead to a complex specification of a fixed *m*(*x*). To simplify the calculations, one can let *m*(*x*) be zero [25–27]:

(4) $$\begin{array}{c} f\left(x_{L,i}\right)\sim Gp\left(0,K\left(x.x^{\prime }\right)\right). \end{array}$$

One can combine Equation (1) and Equation (4) to obtain the prior distribution of *y* [25]:

(5) $$\begin{array}{c} y\sim N\left(0,K\left(x.x^{\prime }\right)+\sigma_{\text{noise}}^{2}I_{n}\right). \end{array}$$

The above equations could be collected as [27]:

(6) $$\begin{array}{c} \left[\underset{\begin{array}{c} f_{l}\\ \underset{f_{T}}{⟶} \end{array}}{⟶}\right]\sim N\left(0,\left[\begin{array}{cc} K\left(x_{L}.x_{L}\right) & K\left(x_{L}.x_{T}\right)\\ K\left(x_{T}.x_{L}\right) & K\left(x_{L}.x_{T}\right) \end{array}\right]\right),\\ \left[\underset{\begin{array}{c} \varepsilon_{l}\\ \underset{\varepsilon_{T}}{⟶} \end{array}}{⟶}\right]\sim N\left(0,\left[\begin{array}{cc} \sigma_{\text{noise}}^{2}I_{n} & 0\\ 0 & \sigma_{\text{noise}}^{2}I_{n} \end{array}\right]\right). \end{array}$$

These equations can be summed up into a Gaussian formulation as [21]:

(7) $$\begin{array}{c} \left[\underset{\begin{array}{c} y_{l}\\ \underset{y_{T}}{⟶} \end{array}}{⟶}\right]\sim N\left(0,\left[\begin{array}{cc} K\left(x_{L}-x_{L}\right)+\sigma_{\text{noise}}^{2}I_{n} & K\left(x_{L}.x_{T}\right)\\ K\left(x_{T}.x_{L}\right) & K\left(x_{L}.x_{y}\right)+\sigma_{\text{noise}}^{2}I_{n} \end{array}\right]\right). \end{array}$$

Then, the Gaussian conditioning rule could be applied to find the posterior distribution of *y*_*T*_, [27]:

(8) $$\begin{array}{c} \left(\left.\overset{⟶}{y_{T}}\right|\overset{⟶}{y_{L}}\right)\sim N\left(\mu_{r},\sum r\right), \end{array}$$

where the mean value and covariance are written as:

(9) $$\begin{array}{c} \mu_{T}=m\overset{⟶}{\left(y_{T}\right)}=K\left(x_{T}.x_{L}\right){\left(K\left(x_{L}.x_{L}\right)+\sigma_{\text{noise}}^{2}I_{n}\right)}^{-1}\overset{⟶}{y_{L}},\\ \sum r=K\left(x_{T}.x_{T}\right)K\left(x_{T}.x_{T}\right)+\sigma_{\text{noise}}^{2}I_{n}-K\left(x_{T}.x_{L}\right){\left(K\left(x_{L}.x_{L}\right)+\sigma_{\text{noise}}^{2}I_{n}\right)}^{-1}K\left(x_{T}.x_{L}\right). \end{array}$$

The theoretical GPR modeling concept is implemented. It is possible to predict the testing dataset outputs through the independent variable and training dataset [28]. These formulations are supportive of the claim that the mean function and covariance could provide a complete GP description through the introduction of the Gaussian distribution. It is important to select a Kernel function (i.e., a strong covariance function) in the training phase. The Kernel matrix has a symmetric, invertible matrix. This contributes to GPR model robustness in target prediction. To identify the optimal Kernel function, the present study manipulated four common Kernel functions, namely, (1) rational quadratic, (2) exponential, (3) squared exponential, and (4) Matern functions, to perform the learning process. The rational quadratic covariance function is defined as:

(10) $$\begin{array}{c} K_{RQ}\left(x.x^{\prime }\right)=\sigma^{2}{\left(1+\frac{x-x^{\prime 2}}{2\alpha \ell^{2}}\right)}^{-\alpha }, \end{array}$$

in which *σ* denotes the amplitude, *σ*^2^ is the variance, *ℓ* represents the length scale, and *α* > 0 is the scale mixture that ascertains the change weights at both small and large scales. The exponential covariance function is formulated as:

(11) $$\begin{array}{c} K_{E}\left(x.x^{\prime }\right)=\sigma^{2}\exp-\left(\frac{x-x^{\prime }}{\ell }\right). \end{array}$$

The squared exponential covariance function is expressed as:

(12) $$\begin{array}{c} K_{SE}\left(x.x^{\prime }\right)=\sigma^{2}\exp\left(-\frac{x-x^{\prime }}{\ell^{2}}\right). \end{array}$$

Finally, the Matern covariance function is represented as:

(13) $$\begin{array}{c} K_{M}\left(x.x^{\prime }\right)=\sigma^{2}\frac{2^{1-v}}{\Gamma \left(\text{v}\right)}\left(\sqrt{2v}\frac{x-x^{\prime }}{\ell }\right)K_{v}\left(\sqrt{2v}\frac{x-x^{\prime }}{\ell }\right), \end{array}$$

where Γ is the gamma function, *K*_*v*_ represents the modified Bessel function, and *ℓ* and *v* are positive variables. In fact, the exponential covariance function and squared exponential covariance function are two particular forms of the Matern covariance function. Setting *v* to 0.5 converts the Matern covariance function into the exponential covariance function. Also, the Matern covariance function transforms into the squared exponential covariance function at a *v* approaching infinity. In light of its additional parameter (i.e., *v*) as a larger degree of freedom, the Matern covariance function could make more accurate estimates as compared to the exponential and squared exponential covariance functions.

## 3. Results and Discussion

### 3.1. Analysis of Validity and Reliability

For the accuracy and reliability evaluation of the developed GPR models in the higher heating value prediction of biodiesels, the present study performed a multivariable statistical test. This work coupled some typical statistical measures and some graphical depictions.

### 3.2. Statistical Variables

For the performance evaluation of the proposed models, the present study exploited the mean square error (MSE), the mean of relative error (MRE), standard deviation (STD), root mean square error (RMSE), and coefficient of determination (*R*^2^).

(14) $$\begin{array}{c} \text{MRE}=\frac{1}{n}\overset{n}{\underset{i=1}{\sum }}\;\frac{\left|y_{\exp.,i}\right.-\left.y_{\text{pred}.,i}\right|}{y_{\text{pred}.,i}},\\ \text{MSE}=\frac{1}{n}\overset{n}{\underset{i=1}{\sum }}{\left(y_{\exp.i}-y_{\text{pred}.i}\right)}^{2},\\ \text{RMSE}=\sqrt{\text{MSE}}=\sqrt{\frac{1}{n}\overset{n}{\underset{i=1}{\sum }}{\left(y_{\exp.i}-y_{\text{pred}.i}\right)}^{2}},\\ \text{STD}=\sqrt{\frac{1}{n-1}\overset{n}{\underset{i=1}{\sum }}{\left(\frac{y_{\exp.i}-y_{\text{pred}.i}}{y_{\exp.i}}\right)}^{2}},\\ R^{2}=1-\frac{\sum_{i=1}^{n}{\left(y_{\text{pred}.i}-y_{\exp.i}\right)}^{2}}{\sum_{i=1}^{n}{\left(y_{\text{pred}.i}-\bar{\;y_{\exp.i}}\right)}^{2}}. \end{array}$$

The statistical parameters related to the mentioned models are calculated and given in Table 1. Dashti and his colleagues used different models to predict the HHV data [19]. The input and output data used in our paper are similar to their work. The most powerful model they presented was the GARBF model, which has ability to estimate the target values with *R*^2^ and MSE equal to 0.9500 and 0.7401, respectively. However, according to the values obtained in Table 1 of our paper, the GPR (exponential) model has the ability to estimate these values with an accuracy of 0.961 and 0.58, respectively.

### 3.3. Point-by-Point Agreement Plot

**Figure 1:**
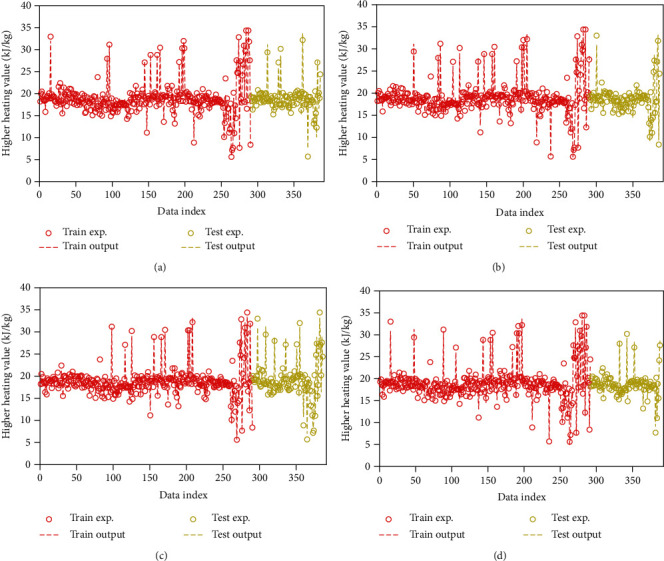
A point-by-point comparison of the modeled estimates to experimental quantities under the (a) exponential, (b) Matern, (c) squared exponential, and (d) rational quadratic kernel functions.

Figure 1 compares the HHV estimates of the GPR models to the measured values, in which “Data Index” represents the sample number, “Train Exp.” Represents the experimental training set, “Train Output” stands for the training set estimate, “Test Exp.” denotes the experimental testing dataset, and “Test Output” represents the testing dataset estimates. According to this figure, most estimates are in good agreement with the experimental data points in all the models. Also, the exponential approach has the highest accuracy and lowest discrepancy. This is supportive of the statistical evaluation findings.

### 3.4. Cross Plot

**Figure 2:**
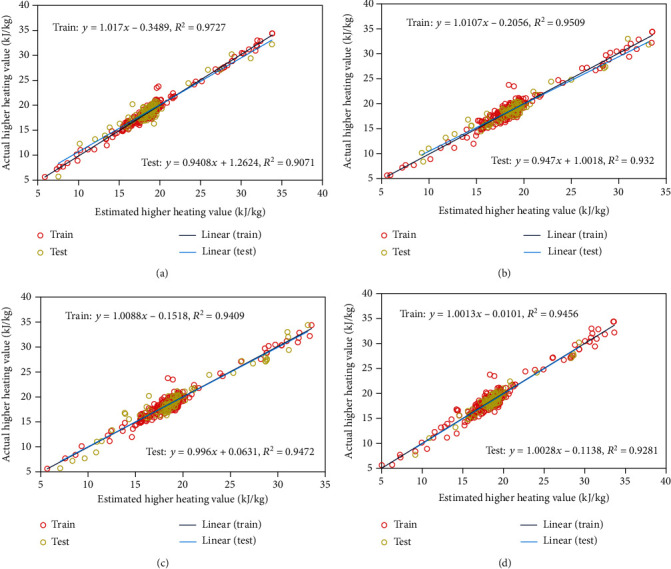
A cross plot comparison of the modeled estimates to the experimental data under the (a) exponential, (b) Matern, (c) squared exponential, and (d) rational quadratic kernel functions.

Figure 2 illustrates the cross plots of experimental HHV quantities versus the corresponding estimates. It further supports the reliability of the proposed models. As can be seen, the linear trend with an *R*^2^ range of 0.90-0.97 demonstrates that the predictions and measurements are consistent for both the training and testing datasets. As can be seen in Figure 2(a), the most accurate results were obtained by the exponential kernel function.

### 3.5. *y* ~ *y*~Relative Deviation Distribution

**Figure 3:**
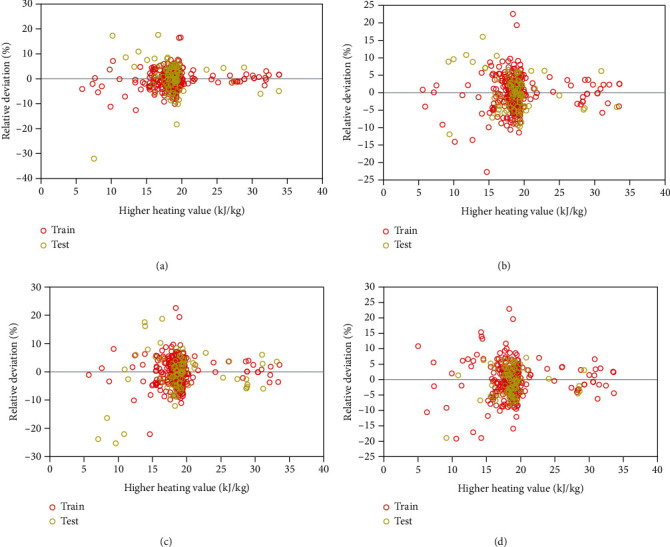
Relative deviations of the GPR models versus HHV measurements under the (a) exponential, (b) Matern, (c) squared exponential, and (d) rational quadratic kernel functions.

Figure 3 depicts the relative deviation distributions of the HHV estimates of the developed GPR models. It should be noted that the relative deviation (RD) is calculated as:

(15) $$\begin{array}{c} \text{RD}\left(\%\right)=100\times \left(\frac{y_{\exp.,i}-y_{\text{pred}.,i}}{y_{\exp.,i}}\right). \end{array}$$

These graphs help determine the degree to which the calculations are realistic based on the experimental quantities. The reliability of the estimates is described by locations of the training and testing data points concerning the horizontal zero-line. According to Figure 3, most relative deviations were found to be from -20% to 20%, which is a favorable range. Furthermore, the points are mostly resting near the horizontal line (Figure 3(a)), in particular those of the exponential kernel function.

### 3.6. Sensitivity Analysis

The present study employed a sensitivity analysis to relate the exponential outputs to the independent input variables. Furthermore, this work employed the relevancy factor (RF) as Pearson's method as [29, 30]:

(16) $$\begin{array}{c} \text{RF}=\frac{\sum_{i=1}^{n}{\left(\bar{x_{k}}-x_{k}.i\right)}^{2}\sum_{i=1}^{n}\left(\bar{y}-y_{i}\right)}{\sqrt{\sum_{i=1}^{n}{\left(\bar{x_{k}}-x_{k}.i\right)}^{2}\sum_{i=1}^{n}{\left(\bar{y}-y_{i}\right)}^{2}}}, \end{array}$$

in which *k* denotes the input type, while *n* represents the number of data points. Also, *x* is the input value, ${\bar{x}}_{k}$ is the average value of input *k*, *y* is the target, and $\bar{y}$ is the average value of the target [31, 32]. RF varies in the range of [-1, 1]; a negative RF represents an inverse relationship between the inputs and output, while a positive RF stands for a direct relationship. A smaller difference between RF and the above-mentioned limits would imply a stronger input-output relationship.

**Figure 4:**
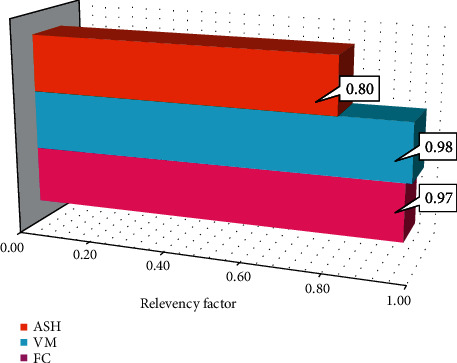
Sensitivity analysis results of the best GPR model.

Figure 4 shows the relative deviation results of the proposed models. As can be seen, all input variables have a direct effect on the HHV. Hence, the proposed models can be said to be able to emulate the effects of several inputs on the target.

### 3.7. Outlier Detection

Laboratory data values are always accompanied by uncertainty. The present work employed the Williams plot of standardized residuals (*R*) versus leverage (*H*) to shed some light on uncertain points. The diagonal entries represent the leverage values in the projection matrix *H* = *X*(*X*^*T*^*X*)^−1^*X*^*T*^, in which *X* represents the explanatory variable matrix, while *T* stands for the transpose matrix operator [33, 34]. A leverage value above the threshold implies uncertainty and a high-leverage point. The leverage threshold is obtained as [35, 36]:

(17) $$\begin{array}{c} H^{*}=\frac{3\left(\text{number of inputs}+1\right)}{\text{number of data points}}. \end{array}$$

**Figure 5:**
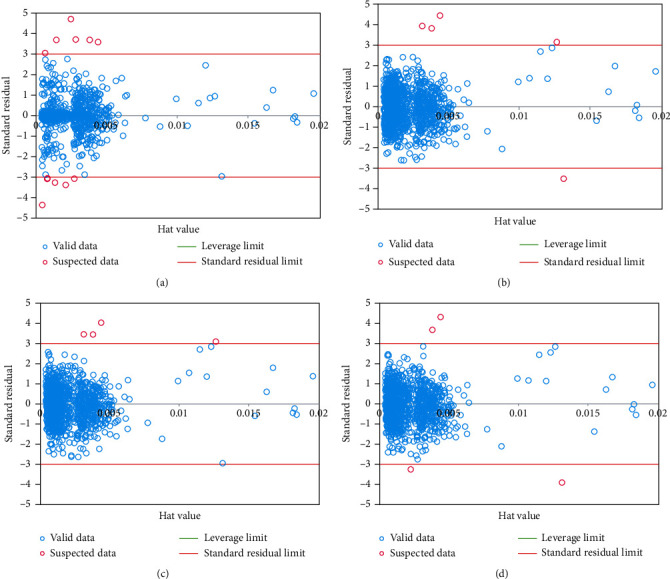
William plots of (a) exponential, (b) Matern, (c) squared exponential, and (d) rational quadratic kernel functions.

Figure 5 illustrates the William plots of the proposed models. One can qualify the data points based on the corresponding locations in the plots. The model applicability domain is represented by the squared area of −3 ≤ *R* ≤ 3 and *H* < *H*^∗^. The area of ≤*R* ≤ 3 and *h* > *H*^∗^ represents the good high leverage data. A question mark represents the model's ability to estimate data points resting in this area. The points that lie in the domains *R* > 3 or *R* < −3 are referred to as the bad high leverage data (i.e., outliers). According to Figure 5, a small number of points exist in the bad high leverage and good high leverage areas; the remaining points fall in the model applicability domain.

## 4. Conclusion

The present study adopted GPR and implemented a comprehensive modeling work on extensive data collected from the literature. HHV was modeled as a function of fixed carbon (FC), volatile matter (VM), and ash *s* by using four Kernel functions. The data were divided into training and testing datasets. This study utilized cross plots, relative deviation diagrams, sensitivity analyses, and Williams plots along with the parametric analysis of errors (including MRE, MSE, RMSE, and *R*^2^). The developed GPR models were found to have high performance in the HHV estimation of biodiesels. The exponential function exhibited the highest accuracy, while the squared exponential function showed the lowest accuracy—the MRE and adjusted *R*^2^ were calculated to be 3.11% and 0.961 for the exponential function, respectively, while they were obtained to be 3.99% and 0.94 for the squared exponential function, respectively. The cross plots and relative deviations demonstrated satisfactory consistency between the HHV measurements and estimates. Finally, the outlier analysis was performed to evaluate data validity and GPR model reliability.

## Figures and Tables

**Table 1:** A comparison of the models in MRE, RMSE, MSE, STD, and *R*-squared for the training, testing, and total data under various Kernel functions.

Model	Phase	*R* ^2^	MRE (%)	MSE	RMSE	STD
GPR (exponential)	Train	0.973	2.70	0.45	0.67	0.46
	Test	0.907	4.53	1.03	1.02	0.63
	Total	0.961	3.11	0.58	1.02	0.52

GPR (Matern)	Train	0.951	3.57	0.78	0.88	0.59
	Test	0.932	4.07	0.80	0.90	0.52
	Total	0.944	3.82	0.83	0.90	0.59

GPR (squared exponential)	Train	0.941	3.40	0.72	0.85	0.58
	Test	0.947	4.91	1.18	1.09	0.69
	Total	0.940	3.99	0.89	1.09	0.62

GPR (rational quadratic)	Train	0.946	3.94	0.92	0.96	0.64
	Test	0.928	3.65	0.59	0.77	0.40
	Total	0.943	3.86	0.84	0.77	0.59

## Data Availability

The data used to support the findings of this study are provided within the paper.
